# A Novel Multiplex Cell Viability Assay for High-Throughput RNAi Screening

**DOI:** 10.1371/journal.pone.0028338

**Published:** 2011-12-05

**Authors:** Daniel F. Gilbert, Gerrit Erdmann, Xian Zhang, Anja Fritzsche, Kubilay Demir, Andreas Jaedicke, Katja Muehlenberg, Erich E. Wanker, Michael Boutros

**Affiliations:** 1 German Cancer Research Center (DKFZ), Division of Signaling and Functional Genomics and Heidelberg University, Department of Cell and Molecular Biology, Heidelberg, Germany; 2 Max Delbrueck Center for Molecular Medicine (MDC), Proteomics and Molecular Mechanisms of Neurodegenerative Diseases, Berlin, Germany; Instituto Nacional de Câncer, Brazil

## Abstract

Cell-based high-throughput RNAi screening has become a powerful research tool in addressing a variety of biological questions. In RNAi screening, one of the most commonly applied assay system is measuring the fitness of cells that is usually quantified using fluorescence, luminescence and absorption-based readouts. These methods, typically implemented and scaled to large-scale screening format, however often only yield limited information on the cell fitness phenotype due to evaluation of a single and indirect physiological indicator. To address this problem, we have established a cell fitness multiplexing assay which combines a biochemical approach and two fluorescence-based assaying methods. We applied this assay in a large-scale RNAi screening experiment with siRNA pools targeting the human kinome in different modified HEK293 cell lines. Subsequent analysis of ranked fitness phenotypes assessed by the different assaying methods revealed average phenotype intersections of 50.7±2.3%–58.7±14.4% when two indicators were combined and 40–48% when a third indicator was taken into account. From these observations we conclude that combination of multiple fitness measures may decrease false-positive rates and increases confidence for hit selection. Our robust experimental and analytical method improves the classical approach in terms of time, data comprehensiveness and cost.

## Introduction

Large-scale RNA-interference (RNAi) screening has become a widely used approach in invertebrate model organisms and in cell culture. RNAi screening has the power to resolve the architecture and dynamic regulation of cellular signalling pathways and can help to identify genetic interactions involved in human diseases [1]. RNAi libraries target almost all annotated genes in the human genome and when used in combination with innovative screening technologies allow the analysis of increasingly complex cellular phenotypes. A common assay type, for example in synthetic lethality screening in cancer addresses the viability or fitness of cells. Synthetic lethality occurs when the combination of a mutation in two different genes results in lethality, whereas when either of the genes is mutated, the organism remains viable. The presence of one of these mutations in e.g. in pathophysiologically altered isogenic or recombinant cells but not in normal cells enables identification of genetic interactions with agents – such as RNAi reagents - that mimic the effect of a second genetic mutation [2]–[6]. Synthetic lethality is indicated by various physiological indicators which are partially and indirectly assessable using fluorescence-, luminescence- or absorbance-based assaying methods. Cellular fitness is often measured by quantifying ATP levels (e.g., CellTiter-Glo), esterase activity and membrane integrity (e.g., Calcein-AM) or by simple cell or nucleus count (e.g. Hoechst DNA stain).

Intracellular ATP [ATP]i serves as an energy carrier that drives virtually all cell functions. Persistent ATP depletion causes a cell to die and in turn cell death is indicated by low ATP levels. Due to its simple accessibility e.g. by an ATP-dependent luciferase-luciferin reaction [ATP]i has been a long-serving indicator of cellular viability. Cellular metabolism creates a continuous demand of energy that requires permanent energy supply. Variation in metabolic activity results in fluctuation of [ATP]i. For example, [ATP]i varies markedly during cell differentiation [7] and with circadian rhythm [8]. It has been reported that genetically identical eukaryotic cells show significant cell-to-cell variability of cellular mitochondrial mass caused by inhomogeneous distribution of mitochondria during cell division [9], which presumably results in varying [ATP]i between cells of the same population. In order for the cell to keep up with fluctuating energy, it employs different metabolic pathways (protein and DNA synthesis, polysaccharide synthesis, and lipid synthesis) which use different trinucleotides (GTP, UTP, and CTP, respectively) as an energy source [6]. While cell death in the long run inevitably results in reduction of [ATP]i, varying [ATP]i is not necessarily an indicator of cell death. Thus, in general [ATP]i is a robust estimator of cell viability, but careful consideration of the mentioned limitations is required when quantification data obtained from [ATP]i measurement are to be analyzed and interpreted.

In healthy cells the cytoplasmic membrane effectively separates the intracellular fluid from the outside environment. It represents an impermeable barrier for charged fluorescent dyes but is permeable for uncharged and hydrophobic compounds such as Calcein acetoxymethyl (AM). Upon permeation of the cytoplasmic membrane, non-fluorescent Calcein-AM is hydrolyzed by intracellular esterases and the product Calcein, a hydrophilic, strongly fluorescent compound remains inside the cell. Thus, Calcein stained cells have esterase activity and in consequence an intact membrane. Due to its low cytotoxicity and simple handling Calcein-AM is a well-suited and commonly employed fluorescent probe for staining viable cells [10]. However, Calcein fluorescence intensity only indirectly reflects membrane integrity by cleavage activity of esterases which depends on cellular metabolism and intracellular ATP. These measurements can often further be complicated by extrusion pumps, which remove fluorophores from the cytoplasm [11]. Hence, in analogy to measuring intracellular ATP for assessment of cellular viability, analysis and interpretation of quantification data requires careful consideration of the mentioned limitations when cellular viability is assessed using indicators of membrane integrity.

Nucleus or DNA stain using fluorescent molecules, such as Hoechst 33342, Hoechst 33258, DAPI or other dyes have been long-serving and commonly applied indicators of cellular viability. Hoechst 33342 exhibits distinct fluorescence emission upon binding into the minor groove of DNA and stains condensed as well as normal chromatin of living or dead cells. The fluorescence intensity of DNA stain is directly proportional to nuclear DNA content and can therefore be used to monitor DNA replication during cell cycle and cell division. When applied to assess cellular viability the total Hoechst fluorescence signal or the number of cell exhibiting DNA stain is quantified [12]. While the simple handling of Hoechst 33342 and similar fluorophores as well as the above described properties of DNA stain enable a broad spectrum of applications, their use in assessment of cellular viability is limited. Due to stoichiometric binding to DNA quantification of total fluorescence signal from cell populations for assessing cellular viability can be misleading. When employed to measure cell number as viability criterion, co-labeling of living and dead cells can yield inaccurate results.

Any of the mentioned approaches yield information on specific cellular conditions and physiological characteristics which only partially and indirectly reflect cellular viability. In fact, none of the described methods clearly and exclusively reflects cellular viability comprehensively. The same is true for other methods assessing cellular viability such as measurement of redox potential by MTT or MTS, the utilization of charged dyes such as trypan blue or propidium iodide or comparable methods. This might explain why striking candidates selected from large-scale screening approaches require re-testing in time-consuming, independently and sequentially conducted secondary experiments to eliminate false-positive hits. To overcome these limitations, we aimed to assess cellular viability by a ‘multiplexing’ approach which combines different methods in a single experiment.

Conventional high-throughput cell-based viability assays, typically run individually and in serial mode, are time and cost intensive and lead to variations. Multiplexing, i.e. the combination of different assaying methods in the same experiment, adds a level of efficiency by reducing sample supply, reducing cell culture and assay consumable requirements. Redundant steps for sample preparation, plate replication, and assay execution are eliminated and experimental variation is reduced.

In this article, we describe a methodology based on a multiplexing approach for synthetic lethality screening to address the issues encountered in the classical assaying methods described above. We aimed to create a viability multiplexing assay which combines multiple indicators in the same experiment, thus decreasing variability and increasing efficiency, throughput and confidence for hit selection. We further aimed to conduct a case study and to measure the quality of the assay using the software *web cellHTS2* [13]. In order to assess the comparability of the employed assaying methods and to calculate false-positive discovery rate, we intended to quantify the overlap of the obtained viability phenotypes.

## Materials and Methods

### Generation of Stable Cell Lines

For generation of stable, tetracycline-inducible HEK-293 cell lines (Invitrogen), Huntingtin poly Q tract of 23 (HTT-Q23, wild type) and poly Q tract of 79 (HTT-Q79, mutant) proteins were subcloned into a gateway compatible pcDNA5/FRT/TO vector, according to the manual of the Flp-In system (Invitrogen) and as reported in [14]. Flp-In HEK-293 host cells contain a single integrated Flp recombination (FRT) site and stably express the tetracycline repressor. Flp-In HEK-293 host cells were cotransfected with the pcDNA5/FRT/TO-Q23 or pcDNA5/FRT/TO-Q79 plasmid respectively and the pOG44 plasmid expressing the Flp recombinase using Lipofectamin 2000 (Invitrogen) following the manufacturer's protocol. Transfected cells were selected with 100 µg/mL Hygromycin B and Blasticidin S for 2 weeks. Five to 10 individual clones were pooled and screened for tetracycline (4 µg/mL) inducible regulation of Q23 and Q79 protein using the Western immunoblotting.

### Cell culture

HEK293 cells were maintained in DMEM (Invitrogen) with 10% fetal bovine serum (Invitrogen) and supplemented with penicillin (100 U/ml)/streptomycin (100 mg/ml) (Invitrogen). Media were additionally supplemented with Hygromycin B (100 µg/mL), Blasticidin S (15 µg/mL), and tetracycline (4 µg/mL).Cell lines were cultured at 37°C, 5% CO_2_ in a humidified incubator according to standard procedures and used for RNAi screening when approximately 80% confluent.

### siRNA transfection

A sub-library of the genome-wide siRNA library siGENOME (Dharmacon, Thermo) targeting the human kinome was used for RNAi screening. The library contained 779 siRNA pools, consisting of four synthetic siRNA duplexes each (dissolved in RNAse free water). Prior to RNAi screening the siRNA pools as well as additional positive and negative siRNA controls with known phenotypes were distributed into white 384-well multititer plates (Greiner) and stored at −20°C until the experiment. The siRNA library was arrayed in multititer plates using a Biomek FX200 liquid handling system (Beckman Coulter). Each well contained 5 µl of a 200 nM pool of four synthetic siRNA duplexes. Library siRNAs were spotted in columns 5–24; the remaining columns were used for controls. Positions E04 and F04 contained a non-targeting siRNA pool as negative control. Positions K04 and L04 contained a siRNA pool targeting WEE1 as positive siRNA transfection control. The gene WEE1 plays a key role in cell cycle and causes cell death when silenced by RNAi [15]. Reverse transfection of cells with siRNA pools was performed by delivering 15 µl of RPMI (Invitrogen) containing 0.05 µl of Dharmafect1 (Dharmacon). After 30 min of incubation at room temperature, 4000 HEK293 cells in 30 µl of DMEM medium (Invitrogen) supplemented with 10% FBS (Invitrogen) were added to the siRNA transfection mix. Plates were incubated for 72 h at 37°C, 5% CO_2_. All dispensing steps were performed with a Multidrop Combi dispensing system (Thermo).

### Cell fitness indicators

#### Calcein-AM

Calcein-AM is a membrane permeable fluorescein derivative able to diffuse into cells. Upon cell entry Calcein–AM is hydrolyzed and thereby modified into the green fluorescent Calcein. This fluorescent signal can be monitored using appropriate filter sets (485 nm excitation/530 nm emission wavelength). Importantly, after modification the molecule remains locally restricted to the cytoplasm and is no longer able to diffuse out of the cell. The fact that Calcein-AM is able to enter only intact cells makes it use to a valuable tool to discriminate viable from dying cells. Thus, the fluorescent signal generated from the assay is directly proportional to the total amount of living cells in a given sample [10].

#### Hoechst 33342

The blue fluorescent Hoechst 33342 (hereafter to be referred to as Hoechst) dye is a cell permeable nucleic acid stain that has multiple applications, including sensitive determination of cell number [15]. The fluorescent signal can be monitored using appropriate filter sets (350 nm excitation/461 nm emission wavelength). The fluorescence of this dye is very sensitive to DNA conformation and chromatin state in cells. Fluorescence is enhanced upon binding to dsDNA at stretches of at least three AT base pairs, but no binding to stretches of two or more GC base pairs. The Hoechst dye requires a [dA-dT]3-[dG-dC]1 sequence to enhance fluorescence, with binding to the bottom of the minor groove as a prerequisite. Hoechst binding to the minor groove of DNA alters chromatin structure [16].

#### CellTiter-Glo Luminescent Cell Viability Assay Kit

The CellTiter-Glo Luminescent Cell Viability Assay is a method to determine the number of viable cells in culture based on quantification of the ATP present, which signals the presence of metabolically active cells. The assay procedure involves adding a single reagent directly to cells which results in cell lysis and generation of a luminescent signal proportional to the amount of ATP present. The amount of ATP is considered to be directly proportional to the number of cells present in culture.

### Fitness multiplexing in RNAi screening

For assessing cell fitness using fluorescence reporters the cells were incubated in 50 µl cell culture media (DMEM) containing 10 µM Hoechst 33342 (Invitrogen) and 10 µM Calcein-AM (Sigma) for one hour at 37°C, 5% CO_2_. Upon fluorescence labelling the staining solution was entirely removed from the wells using a 24-channel wand (VP Scientific) and was replaced by 10 µl DMEM without phenol-red (Invitrogen). Immediately after solution exchange fluorescence intensity of Hoechst stain was measured using a Mithras LB940 plate reader (Berthold Technologies) with 355 nm excitation and 460 nm emission filter set (exposure time: 0.05 s, lamp power: 3000). Subsequently Calcein fluorescence was recorded using the same plate reader with 485 nm excitation and 535 nm emission filter set (exposure time: 0.05 s, lamp power: 3000). The ‘CellTiter-Glo Luminescent Cell Viability Assay Kit’ (Promega) was used according to the manufacturer's instructions and was diluted in DMEM without phenol-red in a ratio of 1∶1. 10 µl of this mixture was added to the cells and incubated for 10 min at room temperature. Subsequently, luminescence intensity was measured using a Mithras LB940 plate reader (no filter, exposure time: 0.1 s). All dispensing steps were performed with a Multidrop Combi dispensing system (Thermo). Screens were performed in duplicate.

### Data analysis

Plate reader data was statistically analyzed using the in-house built software *web cellHTS2*. *web cellHTS2* is a computational tool for statistical analysis of high-throughput screening data sets. The software provides tools for data normalization, quality control, such as correlation analysis, measures of dynamic range of the screening assay as well as scoring (Z-scores) and intuitive visualization of scored screening results. For generation of hit lists quantification data was first normalized to the plate median. Subsequently plate replicates were averaged and cell fitness phenotypes were ranked with lowest mean values indicating strongest phenotypes. Ranked phenotypes from multiplexed cell lines were then subjected to comparative analysis where genes were considered as hits when confirmed by all employed fitness indicators. The full *web cellHTS*2 data sets containing raw values and annotation files are provided in File S1.

## Results

**Figure 1:**
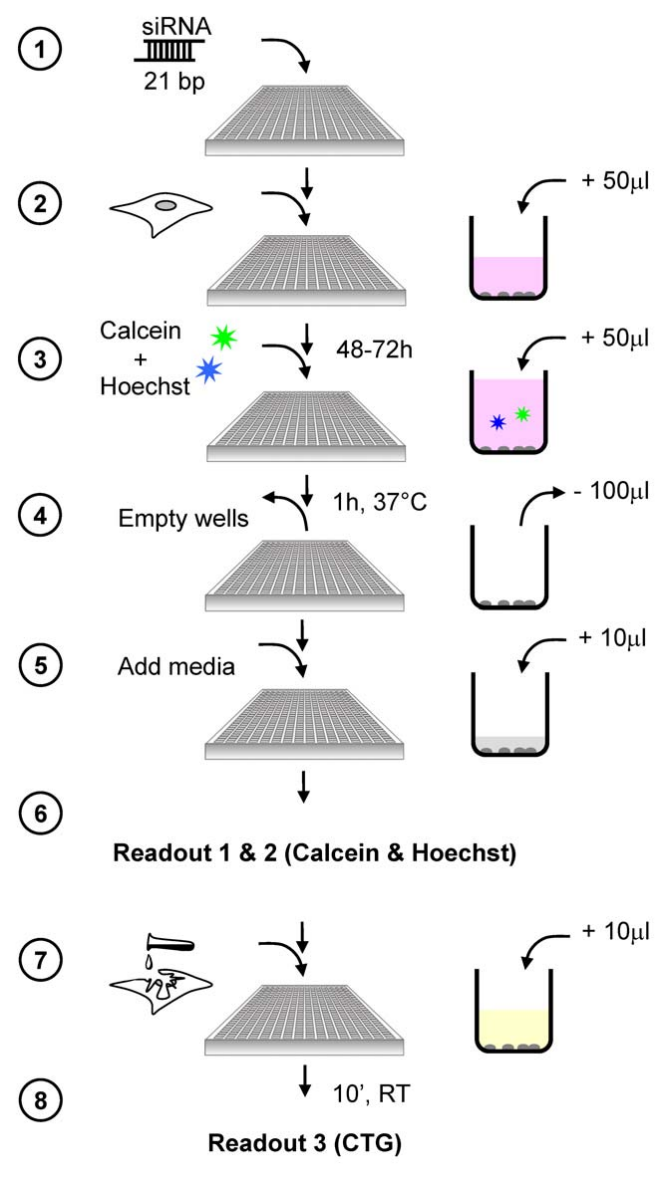
Work flow for cell fitness multiplexing assay. Prior to RNAi screening siRNA pools (Dharmacon) consisting of four individual siRNAs are plated into 384-well multititer plates (1) and stored at −20°C until the experiment. For RNAi screening cells are seeded into the wells of multititer plates at defined density (2) and are reversely transfected for 72 h (5% CO_2_, 37°C). Upon reverse transfection of cells DNA stain Hoechst 33342 and the calcium indicator Calcein-AM are added to the cells in a total volume of 50 µl and incubated for 1 h at 37°C (3). To reduce background fluorescence the staining solution is completely removed from the wells (4) and replaced by 10 µl cell culture media without phenol-red (5). Calcein and Hoechst signals are measured using a plate reader (6). For assessing cell fitness via intracellular ATP level CellTiter-Glo (CTG) is added to the cells and incubated at room temperature for 10 min (7). Luminescence intensity is recorded using a plate luminometer.

We have established an assay for cell fitness multiplexing by combining a biochemical, luminescence-based approach (CellTiter-Glo Luminescent Cell Viability Assay Kit, Promega) and two fluorescence-based assay types (Calcein-AM and Hoechst 33342 DNA stain). The biochemical method assesses cellular fitness by quantifying ATP levels, Calcein labelling reflects cell fitness through membrane integrity and indirect measurement of ATP-dependent enzymatic esterase activity. Hoechst DNA stain correlates cell fitness with cellular DNA content. The experimental workflow is shown in Figure 1.

### Assay development

**Figure 2:**
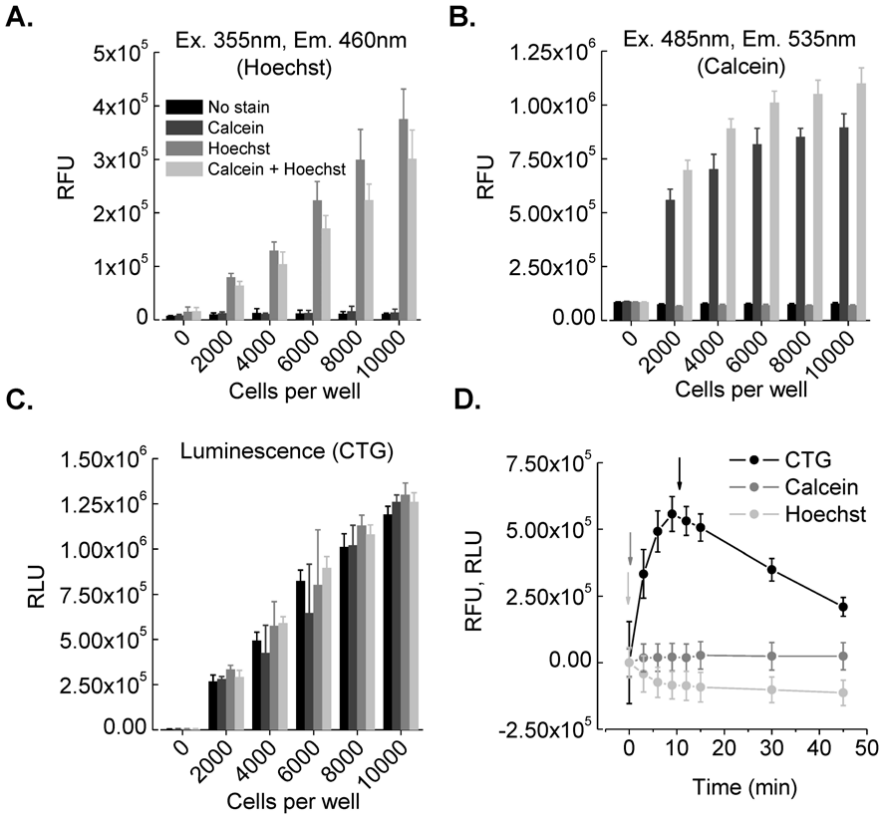
Development of the cell fitness multiplexing assay. **A–C**. Correlation analysis of cell number, fluorescence and luminescence intensities recorded from differently stained HEK293 cells. Cells were seeded at densities between 0–10000 cells per well, cultured overnight and stained with Calcein-AM (dark grey), Hoechst 33342 (grey) and a mixture of Calcein-AM and Hoechst (light grey). For determination of background signal a population of cells remained unstained (black) in the experiments where fluorescence indicators are used. The histograms show averaged fluorescence and luminescence intensities (n = 12 wells, error bars: ±SD). Background signal measured in empty wells is weak compared to the signal measured from stained cells or from unstained cells. Fluorescence intensities of single-stained cells (Hoechst or Calcein) differ significantly from multiplexed cells, probably due to fluorescence quenching of Hoechst by energy transfer to Calcein (Fig. 2B, grey, light grey) and additive excitation of Hoechst by Calcein excitation (Fig. 2C, dark grey, light grey). Luminescence signals are not affected by fluorescence emission. Signal intensities measured in Hoechst (double-stained cells) and CTG fitness indicators (double-stained and CTG treated cells) correlate well to the number of seeded cells whereas fluorescence intensities recorded from double stained cells in the Calcein channel show a non-linear relationship and less correlation. **D**. Time course experiment for determination of optimal timing conditions. Fluorescence intensities (Calcein, grey; Hoechst, light grey) were measured immediately after media exchange (light grey and grey arrows in E., see steps 4–5 in A.) and show either no (Calcein) or only weak (Hoechst) variation over time. Luminescence signals (black) reach a maximum ten minutes after addition of CTG (see step 7 in Fig. 1A) followed by linear decrease. The black arrow indicates the optimal time point for luminescence measurement. To avoid strong variance of measured luminescence signals this time point is crucial and needs strict obedience. Data were averaged from 12 wells per condition, error bars: ±SD.

To evaluate the correlation of cell number to fluorescence/luminescence intensity we seeded HEK293 cells at densities between 0–10000 cells per well into white 384-well plates and cultured the cells overnight. The next day cells were loaded with Calcein-AM, with Hoechst 33342 and with a mixture of Calcein AM and Hoechst 33342 for 1 h at 37°C. For determination of cellular background fluorescence a population of cells remained unstained. Upon loading of fluorescence indicators the staining solution was completely removed from the wells and was replaced by cell culture media without phenol-red to reduce light scattering and background fluorescence. In this configuration Calcein and Hoechst signals were measured sequentially using a plate reader. For assessing cell fitness via intracellular ATP level CTG was added to the cells and luminescence intensity was recorded using the same plate reader (see methods section for details). Histograms in Fig. 2 show averaged fluorescence intensities (n = 12 wells, error bars: ±SD) measured at 460 nm (Hoechst, Fig. 2A), at 535 nm (Calcein, Fig. 2B) and without filter (luminescence, Fig. 2C). Background signal measured in empty wells is weak compared to the signal measured from stained cells (4000 cells/well, 12.5%, 11.7% and 0.5% for Calcein, Hoechst and CTG respectively) or from unstained cells (4000 cells/well, 11.1% and 9.9% for Calcein and Hoechst respectively). Fluorescence intensities of single-stained cells (Hoechst or Calcein-AM) differ significantly from multiplexed cells (Hoechst, single: 129349±16406, multiplexed: 104003±22899; Calcein, single: 701597±69538, multiplexed: 890121±45038), probably due to fluorescence quenching of Hoechst by energy transfer to Calcein (Fig. 2B, grey, light grey) and additive excitation of Hoechst by Calcein excitation (Fig. 2C, dark grey, light grey). Luminescence signals are not affected by fluorescence emission. Signal intensities measured in Hoechst (double-stained cells) and CTG fitness indicators (double-stained and CTG treated cells) correlate well to the number of seeded cells (Hoechst 33342, R^2^ = 0.99±0.44; CTG, R^2^ = 0.99±1.3) whereas fluorescence intensities recorded from double stained cells in the Calcein channel show a non-linear relationship and less correlation (R^2^ = 0.95±5.4).

To determine the optimal timing conditions for fluorescence and luminescence recordings we conducted time course experiments with the individual cell fitness assays. For that HEK293 cells were seeded at a density of 10000 cells per well, incubated overnight and subsequently prepared for cell fitness multiplexing as shown in Fig. 1. Fluorescence and luminescence intensities were recorded at eight different time points for a total of 45 min (0, 3, 6, 9, 12, 15, 30, 45 min). Fig. 2D shows the time courses for Calcein and Hoechst fluorescence emission as well as luminescence signal (CTG). Fluorescence intensities show either no (Calcein, Fig. 2D, dark grey) or only weak (Hoechst, Fig. 2D, light grey) variation over time. Luminescence signals (CTG, Fig. 2D, black) reach a maximum 10 min after addition of CTG followed by a linear decrease. The arrows in Fig. 2D indicate the optimal time points for fluorescence and luminescence measurements. To avoid strong variance of measured luminescence signals this time point is crucial and needs strict obedience. Data were averaged from twelve wells per condition (error bars: ±SD).

### RNAi screening

**Figure 3:**
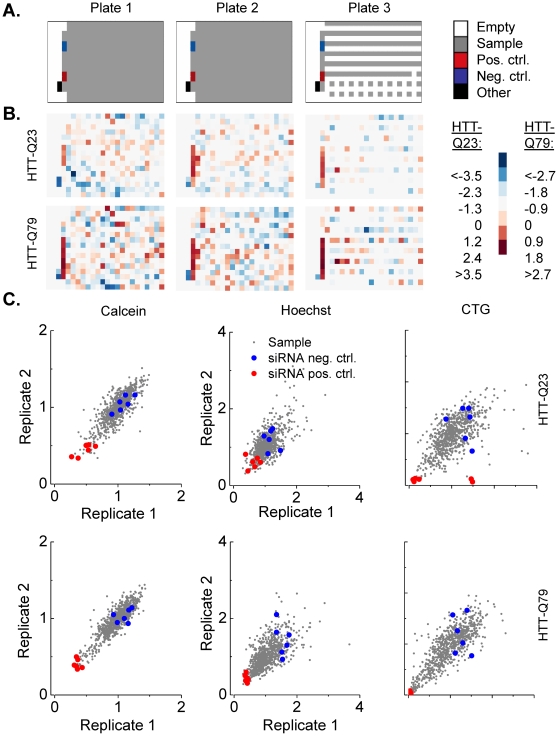
Proof-of-principle and quality control of the viability multiplexing assay. Two cell lines stably expressing wild type (HTT-Q23) and mutant Huntingtin protein (HTT-Q79) were transiently and reversely transfected with siRNA-pools (Dharmacon) targeting the human kinome additional siRNA controls. Each siRNA-pool was transfected in two replicates, incubated for 72 h and subsequently prepared for viability multiplexing as shown in Fig. 1 **A**. Configuration of 384-well plates for RNAi screening. **B**. Image plots of scored values from CTG readout with two cell lines. **C**. Correlation analysis of two replicates each for both cell lines measured in all fitness indicators. Data were normalized to the plate median. Grey dots represent sample siRNAs and siRNA controls are shown in colors (positive, red; negative, blue). Coefficients of determination (R^2^) for all fitness indicators and cell lines are summarized in Table 1.

In a preparatory step prior to RNAi screening pre-distributed and frozen siRNA pools (see Fig. 3A for pipetting scheme) were thawed at room temperature for 30 min. Two cell lines stably expressing wild type (HTT-Q23) and mutant Huntingtin protein (HTT-Q79) were reverse-transfected with siRNA-pools in two replicates, incubated for 72 h and subsequently prepared for cell fitness multiplexing as shown in Fig. 1. Quantification data was statistically analyzed using the software *web cellHTS2*. Fig. 3B shows image plots of scored values (Z-scores) calculated by *web cellHTS2*. The color-coded representation facilitates fast and intuitive interpretation of screening results even from large data sets where hundreds of multititer plates are used in the same experiment. Warm colours (red) indicate large Z-scores and decreased viability whereas cold colours (blue) and low Z-scores represent unaffected or increased viability relative to the median of all samples per 384-well plate. The image plots indicate low sample variation and excellent performance of functional positive and negative siRNA controls. To assess replicate reproducibility we analysed replicate correlation for both cell lines measured with three fitness indicators. Fig. 3C shows correlation plots for replicated data normalized to the plate median. Grey dots represent sample siRNAs and siRNA controls are shown in colors (positive: red; negative: blue). Coefficients of determination (R^2^±SD) for all fitness indicators and cell lines are summarized in Table 1. The highest replicate correlation was determined for the fluorescence indicator Calcein (HTT-Q23: 0.82±0.11, HTTQ79: 0.88±0.09), followed by the biochemical approach CellTiter-Glo (HTT-Q23: 0.66±0.26, HTT-Q79: 0.81±0.21) and nucleus count using the fluorescence indicator Hoechst 33342 (HTT-Q23: 0.65±0.25, HTT-Q79: 0.65±0.28). The overall high replicability of the employed indicators implies a good quality of the assay and its suitability for high-throughput screening.

**Table 1:** Summary of squared correlation coefficients (R^2^±SD) of both replicates from large-scale RNAi screen with three cell lines read in viability multiplexing experiment.

Cell line	Calcein	Hoechst	CTG
HTT-Q23	0.82±0.11	0.65±0.25	0.66±0.26
HTT-Q79	0.88±0.09	0.65±0.28	0.81±0.21

### Selection of fitness phenotypes

**Figure 4:**
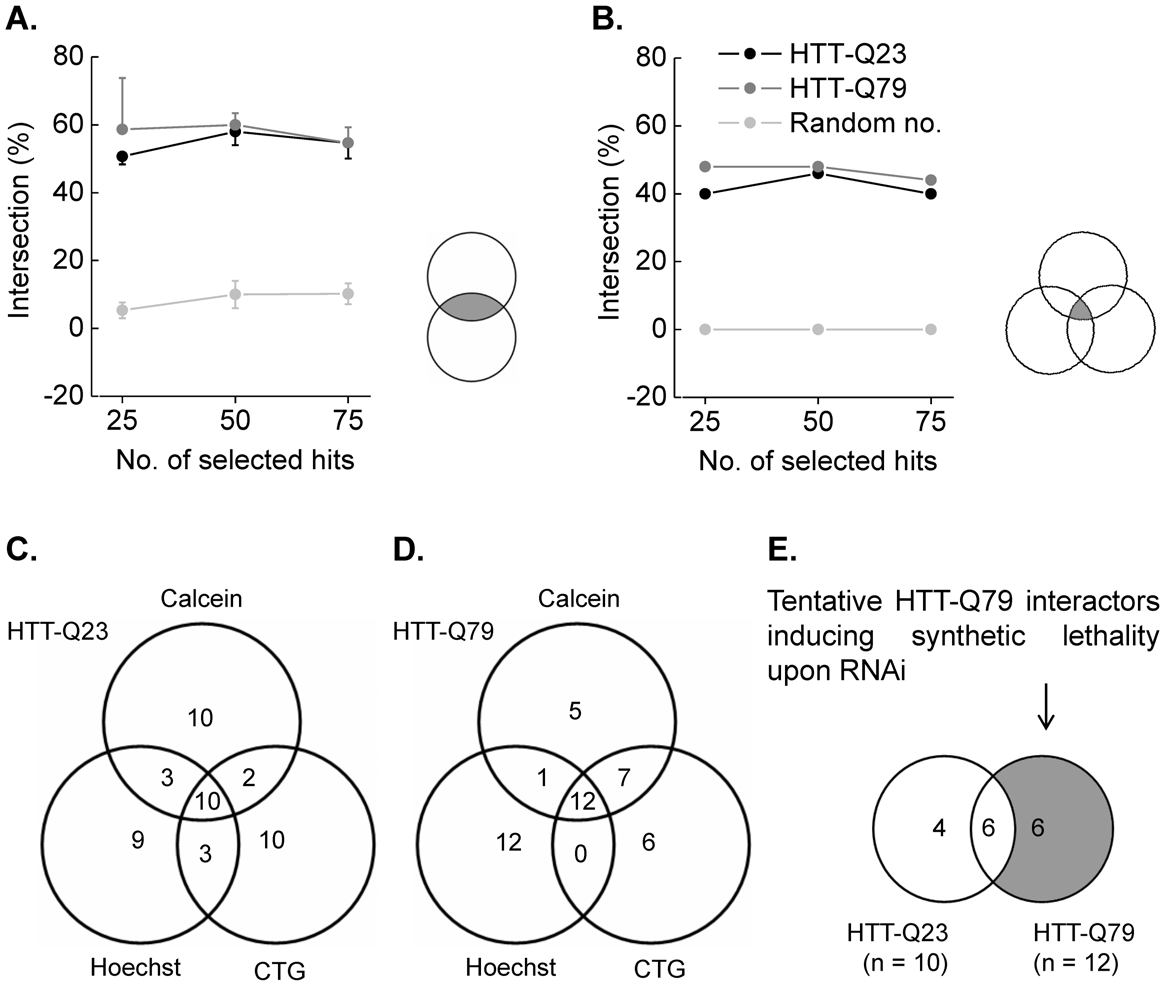
Analysis of fitness multiplexed cell lines and identification of cell line-specific fitness phenotypes. **A–B**. Hit confirmation by combinatorial analysis of multiple fitness indicators. Recorded fluorescence and luminescence intensities were normalized to the plate median and plate replicates were averaged for further analysis. Cell fitness phenotypes were selected from ranked mean values with small values indicating strong decrease in fitness. 25, 50 and 75 top-ranking fitness phenotypes were selected for both cell lines as well as for randomly generated values from all fitness indicators and the intersection (in %) of phenotypes identified in two (**A**.) and three (**B**.) channels was calculated as measure of hit confirmation. The fraction of intersecting phenotypes from two or three channels is comparable for both cell lines and differs significantly from randomly generated numbers. These results indicate that the fitness indicators are strongly linked and that combination of multiple fitness readouts increases confidence for hit selection. **C–D**. Venn-diagrams for the three fitness indicators constructed from selected top 25 fitness phenotypes of both cell lines. **E**. Identification of cell-line specific fitness phenotypes. To identify genes relevant to HD from the proof-of-principle study we identified siRNAs which showed strong cell fitness effects in the Huntingtin mutant cell line (HTT-Q79) but not in wild type cells (HTT-Q23). A total of 6 siRNAs were identified (see grey area in overlapping circles).

Recorded fluorescence and luminescence intensities were normalized to the plate median using *web cellHTS2* and plate replicates were averaged for further analysis. Cell fitness phenotypes were selected from ranked mean values with small values indicating strong decrease in fitness. The top-ranking 25, 50 and 75 fitness phenotypes were selected for both cell lines from all fitness indicators as well as for randomly generated values and the intersection (in %) of hits identified in two or three channels was calculated as measure of hit confirmation (see Fig. 4A, B). The intersection of two (see grey area in two overlapping circles in Fig. 4A) and of three channels (see grey area in three overlapping circles in Fig. 4B) was calculated for all channel combinations and was averaged for visualization. The fraction of intersecting phenotypes from two (Fig. 4A, HTT-Q23, grey: 50.7±2.3% (25), 58.0±4.0% (50), 54.7±6.4% (75); HTT-Q79, dark grey: 58.7±15.1% (25), 60.0±3.5% (50), 54.7±6.4% (75)) or three channels (Fig. 4B, HTT-Q23, grey: 40% (25), 46% (50), 40% (75); HTT-Q79, dark grey: 48% (25), 48% (50), 44% (75)) is comparable for both cell lines and differs significantly from randomly generated numbers (light grey, Fig. 4A: 5.3±2.3% (25), 10.0±4.0% (50), 10.2±3.1% (75); Fig. 4B: 0 (25, 50, 75)). Venn-diagrams for the three fitness indicators constructed from selected top 25 fitness phenotypes of both cell lines are shown in Fig. 4C, D.

These results show that a cell fitness phenotype is not necessarily and equally represented by different fitness indicators, it is rather represented by a variety of effects which can only partially be assessed by individual fitness indicators. To a certain extend the assayed fitness indicators are physiologically linked which is indicated by the comparable overlap of identified cell fitness phenotypes. Multiplexing of various physiologically linked fitness indicators therefore results in a decreased number of fitness phenotypes on the one hand but an increased fraction of repeatedly confirmed phenotypes on the other hand.

### Case study: identification of genes involved in Huntington's disease

Huntington's disease (HD) is a progressive neurodegenerative genetic disorder, which affects muscle coordination and leads to cognitive decline and dementia. The disease is caused by an autosomal dominant mutation in either of an individual's two copies of a gene called Huntingtin (HTT). The human Huntingtin gene consists of 67 exons which encode a protein of 3144 amino acids and is ubiquitously expressed throughout all human tissues. Although its cellular functions are not yet fully understood, Huntingtin has been implicated in transcription, apoptosis, lipid metabolism, Wnt signalling, trafficking, mitochondria and calcium homeostasis, cell cycle and other cellular processes [17]–[29]. Expansion of a CAG tri-nucleotide repeat in exon 1 of the Huntingtin gene above a critical threshold of 35–40 CAG repeats causes HD. The mutation encodes an expanded polyglutamine (poly Q) tract which renders the mutant poly-peptide prone to aggregation [30]. We have generated stably transfected human HEK293 T-Rex cell lines that upon tetracycline-induction conditionally express Huntingtin poly Q tract of 23 (HTT-Q23, wild type) and poly Q tract of 79 (HTT-Q79, mutant), respectively [14].

While recombinant expression of mutant HTT protein revealed no difference in viability compared to cells expressing the wild type protein (data not shown) we suspected that a second mutation, mimicked by gene silencing, might cause synthetic lethality, thus uncovering mutant HTT specific genetic interactions. We therefore analysed the loss-of-function phenotype of genes encoding human kinases in both recombinant cell lines. To identify mutant HTT protein-specific synthetic lethality we first selected 25 top-ranking genes from the three assaying methods and calculated the intersection of co-occurring viability phenotypes. From 25 selected top-ranking fitness phenotypes a total of 10 (40%) in the HTT-Q23 cell line and 12 (48%) in the HTT-Q79 cell line were parallel identified by all fitness indicators. In a next step we analyzed the intersection of fitness phenotypes in both recombinant cell lines (see Fig. 4C, D) and chose those siRNAs, which induced cell fitness effects in the mutant cell line (HTT-Q79) but not in Huntingtin wild type cells (HTT-Q23). A total of six siRNA pools were identified as shown in Fig. 4E (see grey area in overlapping circles). The gene PI3K was reported to be involved in regulating glucose uptake and metabolism [31]. The gene WEE1 plays a key role in cell cycle and the gene FLJ10761 encodes the protein ethanolamine kinase 2 which catalyzes phosphatidylethanolamine biosynthesis. WEE1 and FLJ10761 were reported to be associated with Alzheimer's disease [32], [33]. The gene MAPK8 was reported to be involved in Huntington's disease [34] and the genes WIF and CSNK2A2 are known to participate in the Wntpathway [35], [36] which was reported to be linked to HD [19], [20].

None of the identified genes were further confirmed by re-testing with multiple independent siRNAs in independent assays. Thus, the identified genes are being considered as ‘primary hits’ hits - in terms of the classical approach where only a single indicator is assessed - until validation by functional analysis.

### Comparability of assaying methods

To assess the comparability of the employed fitness indicators we calculated the average overlap of 25 top-ranking phenotypes identified by two and by three assaying methods. 50.7±2.3% (HTT-Q23) and 58.7±14.4% (HTT-Q79,) of the selected viability phenotypes were confirmed by a second fitness indicator. When a third readout channel was included in the analysis the percentage of co-occurring fitness phenotypes decreased to 40% (HTT-Q23) and 48% (HTT-Q79). All of the employed detection channels are comparable for reproducing hits as described above and as shown in Fig. 4C, D. The reported decrease in co-occurring fitness phenotypes thus cannot be ascribed to poor hit reproducibility of a single viability indicator. In fact, the observed phenotype intersections indicate that a large proportion of the identified phenotypes are wrongly identified hits, or false-positive hits. False-positive hit rates range between 39.7–41.3% for a single readout-based viability assay and increase up to 52–60% in the double readout-based approach. Following the terminology of hit classification false-positive hits are to be considered as true-negative hits and phenotypes confirmed by a second (HTT-Q23: 50.7±2.3%; HTT-Q79: 58.7±14.4%) or third (HTT-Q23: 40%, HTT-Q79: 48%) fitness indicator are thus to be considered as true-positive hits. Phenotypes excluded from the list of 25 top-ranking genes are considered as negatives which were not further analysed. According to this, the proportion of false-negative hits was not quantified and remains unknown.

## Discussion

For the sake of feasibility, throughput and cost reasons, cell-based viability or fitness screening in high-throughput mode is often conducted by single channel readout approaches with the result of limited reliability regarding the confidence of identified genes and false-positive discovery rate. The initial benefit is often compromised by subsequent time-consuming and cost-intensive re-screens for hit-confirmation and validation. To address this issue, we have developed a multiplexing approach for assessment of cellular viability that is cost-effective, reliable and that is advantageous for high-throughput fitness screening for several reasons. First, this assay is low-, medium- to high-throughput compatible, thus allowing for efficient and thorough screening. Second, the approach makes use of easy-to-use reagents, circumventing the need for more complicated assays such as those involving transfection plasmids encoding for luminescence-based fitness indicators. Third, this assay reveals robust phenotypes confirmed by comparative analysis of multiple readout channels.

We applied the assay in a large-scale RNAi screening experiment with siRNA pools targeting the human kinome in HEK293 cells expressing wild type and mutant Huntingtin protein and we identified a number of synthetic lethal genetic interactions relevant to Huntington's disease. None of the identified genes were further confirmed by re-testing with multiple independent siRNAs in independent assays. One gene (MAPK8) was reported to be directly associated with Huntington's disease [34]. Two genes (WIF, CSNK2A2) were shown to participate in Wnt-signaling [35], [36] which was reported to be involved in HD [19], [20]. Half of the identified genes exhibit no direct or indirect association to HD, although the genes FLJ10761, WEE1 and PI3K had previously been linked to neurodegeneration in Alzheimer's disease and lipid metabolism that was reported to be affected in HD [31]–[33]. The high rate of identified HD-related genes is the result of a thorough and efficient elimination of false-positive hits.

Our comparative analysis revealed a false-positive discovery rate of 39.7–41.3% for the single readout-based assay and a rate of 52–60% for a double readout-based assaying approach that is conducted using three different viability indicators. These results show the limitation of the classical approach and strongly imply that single channel measurements comprise a substantial level of uncertainty.

The observed degree of phenotype intersection in comparative analysis is reproducible and comparable between the employed fitness marker and the reported decrease in co-occurring phenotypes along with increasing number of parallelized measures cannot be ascribed to poor hit reproducibility of a single viability indicator. In fact, the large proportion of identified false-positive hits meets the expectation for the employed fitness markers quite well. The applied probes are strongly biased when applied to quantify cell fitness as they assess different biological properties. The indicators yield information on specific cellular conditions and physiological characteristics which only partially and indirectly reflect cellular viability. CellTiter-Glo assesses intracellular ATP content, Calcein fluorescence reflects ATP-dependent esterase cleavage activity and Hoechst stain indicates nuclear DNA content. None of the used indicators clearly and exclusively reflect cellular viability comprehensively. The high degree in false-positive discovery rate is therefore an expected result of the individual markers that presumably represent real biological differences. In another experimental context, this probe-specific and additional information which was not further analyzed in the present study could be exploited in more detail to add a level of biological content besides assessment of cellular viability.

Our results suggest that the extension of the classical approach by implementation of a second indicator is sufficient to significantly increase quality and robustness of screening data. The application of a third viability marker further improves confidence for hit selection and helps to remove false-positives but the advantage is less striking and the cost-benefit ratio needs careful consideration when applied routinely. In the context of our proof-of-concept study, conducted to evaluate the comparability of different viability indicators, employment of a third fitness marker was beneficial and helped to identify synthetic lethal genetic interactions relevant to in HD. Presumably, such an extension is advantageous for a multitude of other screening projects but implementation of additional probes for multiplexing could also be compromising or disadvantageous. For example when screening costs and preparation and reading time of existing assays increase disproportionality. The CellTiter-Glo reagent is expensive compared to Hoechst DNA stain or Calcein-AM ester and can dramatically increase assay reagent expenditures and large-scale screening approaches using libraries that contain numerous samples would of course be affected to a higher degree than low to medium-throughput experiments. Another aspect that needs careful consideration when employing multiple indicators is potential perturbation of primary fluorescent or luminescent probes by chemical or optical interference. Thus, careful evaluation and optimization upon implementation of additional indicators is strongly suggested to avoid unexpected impairment of established assaying techniques. The decision of whether a third indicator is advantageous for an existing screening approach strongly depends on the individual assay conditions, the biological question to be addressed and of course the available instrumental infrastructure and, hence, can only be made in case-specific manner and upon careful consideration of the mentioned criteria.

In summary, our experimental and analytical method improves the classical cell viability assaying approaches in terms of time, data comprehensiveness and cost. Based on our observations it is very tempting to state that fitness multiplexing and subsequent comparative analysis of multi-parametric data enables accelerated hit confirmation compared to the classical approaches and eliminates the need for additional re-testing using independent assays. Due to the fact that the employed fitness indicators assess different biological properties, they also provide supplementary biological content that was discarded in the present study by exclusion of false-positive hits but that could be exploited in another biological context. It is important to mention that the applicability of the described method depends on the individual experimental setup, on the number of samples to be tested, the available instrumental infrastructure and the biological question to be assessed.

While this work focuses on RNAi screening, our method could also be adapted for other strategies, such as small molecule or combined RNAi and small molecule screening. Altogether, this work contributes to furthering the applicability of cell-based high-throughput fitness screening.

## Supporting Information

File S1 The folder ‘Archive S1.7z’ contains all raw data and annotation files used for this manuscript.
(7z) Click here for additional data file.
